# miR-33a Inhibits the Differentiation of Bovine Preadipocytes through the IRS2–Akt Pathway

**DOI:** 10.3390/genes14020529

**Published:** 2023-02-20

**Authors:** Wenzhen Zhang, Sayed Haidar Abbas Raza, Bingzhi Li, Bing Sun, Sihu Wang, Sameer D. Pant, Nouf S. Al-Abbas, Nehad A. Shaer, Linsen Zan

**Affiliations:** 1 College of Animal Science and Technology, Northwest A&F University, Yangling 712100, China; 2 Safety of Livestock and Poultry Products, College of Food Science, South China Agricultural University, Guangzhou 510642, China; 3 Gulbali Institute, Charles Sturt University, Boorooma Street, Wagga Wagga, NSW 2678, Australia; 4 Department of Biology, Jamoum University College, Umm Al-Qura University, Makkah 21955, Saudi Arabia; 5 Department of Chemistry, Al Lieth University College, Umm Al-Qura University, Makkah 21955, Saudi Arabia; 6 National Beef Cattle Improvement Center, Northwest A&F University, Yangling 712100, China

**Keywords:** miR-33a, bovine, preadipocyte differentiation, *IRS2*, Akt pathway

## Abstract

Several microRNAs (miRNAs) are known to participate in adipogenesis. However, their role in this process, especially in the differentiation of bovine preadipocytes, remains to be elucidated. This study was intended to clarify the effect of microRNA-33a (miR-33a) on the differentiation of bovine preadipocytes by cell culture, real-time fluorescent quantitative PCR (qPCR), Oil Red staining, BODIPY staining, and Western blotting. The results indicate that overexpression of miR-33a significantly inhibited lipid droplet accumulation and decreased the mRNA and protein expression of adipocyte differentiation marker genes such as peroxisome proliferator-activated receptor gamma (PPARγ), sterol regulatory element-binding protein 1 (SREBP1), and fatty acid-binding protein 4 (FABP4). In contrast, the interference expression of miR-33a promoted lipid droplet accumulation and increased the expression of marker genes. Additionally, miR-33a directly targeted insulin receptor substrate 2 (*IRS2*) and regulated the phosphorylation level of serine/threonine kinase (*Akt*). Furthermore, miR-33a inhibition could rescue defects in the differentiation of bovine preadipocytes and the Akt phosphorylation level caused by small interfering *IRS2* (si-IRS2). Collectively, these results indicate that miR-33a could inhibit the differentiation of bovine preadipocytes, possibly through the IRS2–Akt pathway. These findings might help develop practical means to improve the quality of beef.

## 1. Introduction

Adipose tissue is metabolically active, modulating host feed intake and metabolism in response to energy variations, and is consequently regarded as an endocrine organ [1,2]. Recent studies have demonstrated that adipose tissue plays a critical role in systemic metabolic health and that adipocyte disruption can cause many metabolic diseases in primary functions, such as lipid storage, endocrine function, and responsiveness to insulin [3]. In humans, excess adiposity can lead to obesity, which in turn is linked to a variety of other diseases such as type 2 diabetes, hepatic steatosis, cardiovascular diseases, and a multitude of cancers [4,5,6,7]. Apart from health, in livestock species, the deposition of adipose tissue can influence key indicators of meat quality such as the intramuscular fat and lean meat ratio, which can impact the productivity and profitability of livestock farming, particularly those involved in meat production [8]. Adipose tissue expansion is driven by hyperplasia, in which preadipocytes form new adipocytes through adipogenesis, and hypertrophy, in which tissue size increases; both of these processes occur during normal growth, as well as in obesity development [9,10]. Hyperplasia and hypertrophy are highly complex and tightly regulated processes involving many transcription factors, including PPARγ, SREBP1, and FABP4, which are essential for adipocyte differentiation and the regulation of adipogenesis [11,12,13]. Many genes have been identified as direct targets of miRNAs in adipogenesis [14].

miRNAs are highly conserved short non-coding RNA molecules comprised of approximately 22 nucleotides. They are key constituents of RNA induced silencing complexes (RISC) that allow RISCs to bind to and target specific messenger RNA molecules, thereby enabling transcriptional or post-transcriptional regulation of gene expression [15]. MiR-143 and miR-17-92 were initially thought to have significant regulatory roles in adipose tissue differentiation of several species [16,17,18]. In the following decades, many miRNAs were reported to be implicated in the proliferation and differentiation of preadipocytes, including miR-222, miR-145, miR-424, and miR-378 [19,20,21]. In previous bovine studies, castration was found to increase the amount of intramuscular adipose tissues, and subsequent transcriptome sequencing demonstrated that miR-33a expression was significantly upregulated in the intramuscular adipose tissues of bulls when compared with steers (GEO: GSE75063) [22]. Our previous study further supports the hypothesis that miR-33a likely impedes the proliferation of bovine preadipocytes by targeting the *CDK6* gene [23]. However, the effects of miR-33a on the differentiation of bovine preadipocytes remain unclear.

A transcriptome-sequencing-based study has shown that miR-33a overexpression in bovine preadipocytes leads to differential expression of genes associated with the PI3K–Akt signaling pathway (Accession: PRJNA783618) [23]. It is well known that the PI3K–Akt signaling pathway is an essential regulator of growth, metabolism, and survival of normal cells [24], which also plays a crucial role in the differentiation of preadipocytes [21]. Studies have shown that *IRS1/2* could activate PI3K–Akt to regulate the expression of *PPARγ* and ultimately affect the proliferation, differentiation, and apoptosis of preadipocytes [25,26]. Studies have also shown that *IRS1* and *IRS2* have essential functions for regulating cellular processes such as growth, development, survival, and metabolism [27]. *IRS2* has also been shown to promote the differentiation of human bone marrow-derived mesenchymal stem cells into adipocytes [28]. Moreover, the dual knockdown of *IRS1* and *IRS2* can impair 3T3-L1 preadipocyte differentiation [29]. Taken together, we speculate that miR-33a likely regulates the differentiation of bovine preadipocytes through the IRS2–Akt pathway.

## 2. Methods

### 2.1. Ethics Statement

All of the animal handling procedures used in this study were approved by the Committee of Experimental Animal Management at Northwest Agriculture and Forestry University, China (protocol number: NWAFUCAST2018-168).

### 2.2. Sample Preparation, Cell Culture, and Transfection

Bovine preadipocytes were isolated from the perirenal adipose tissues of three healthy one-day-old Qinchuan bulls, as previously described [30]. Briefly, perirenal tissue samples were subjected to three rounds of rinsing with phosphate-buffered saline (PBS; Hyclone, Logna, UT, USA) supplemented with 10% antibiotics (penicillin/streptomycin) and digested with I Collagenase in a 37 °C shaking water bath for 1–2 h. A cell sieve was used to filter the cells following digestion, and they were then centrifuged. The supernatant was subsequently discarded, and finally, the cells were seeded on plates.

Bovine preadipocytes were cultured in DMEM/F12 (Hyclone, USA) supplemented with 10% fetal bovine serum (FBS; PAN-Biotech, Adenbach, Germany) and 100 U/mL penicillin/streptomycin (Hyclone) with cell culture plates in 37 °C and 5% CO_2_ incubators. At 100% confluence, bovine preadipocytes induced adipogenic differentiation in DMEM/F12 supplemented with 10% FBS, 100 U/mL penicillin/streptomycin, 0.5 mM 3-isobutyl-1-methylxanthine (IBMX), 1 mM dexamethasone, and 10 μg/mL insulin. After two days, the culture medium was changed to DMEM/F12 supplemented with 10% FBS, 100 U/mL penicillin/streptomycin, and 10 μg/mL insulin. At 80% confluence, the bovine preadipocytes were transfected with 50 nM miR-33a mimic, 50 nM mimic negative control (NC), mimic NC (Cy3), 100 nM miR-33a inhibitor, and 100 nM inhibitor NC using Lipofectamine 3000 reagent (Thermo Fisher Scientific, Waltham, MA, USA) according to the manufacturer’s instructions. After reaching near 100% confluence, the bovine preadipocytes were induced to differentiate into adipocytes. Mimic NC (Cy3) was used to identify the effects of the transfection.

### 2.3. RNA Preparation and Quantitative Real-Time PCR

RNAiso Plus (Takara, Dalian, China) was used to extract the total RNA from about 2 × 10^7^ bovine preadipocytes according to the manufacturer’s instructions. Reverse transcription and qRT-PCR of miRNA and mRNA were performed as described previously [31]. In brief, each qRT-PCR reaction solution for mRNA consisted of the following: 12.5 μL TB Green Premix Ex Taq (Tli RNaseH Plus) (2 X) 0.5 μL PCR Forward Primer (10 μM), 0.5 μL PCR Forward Primer (10 μM), DNA template (<100 ng), and Rnase Free dH2O (up to 25 μL). The three steps of reaction conditions were as follows. Step 1: 95 °C for 30 s. Step 2 (40 cycles): 95 °C for 5 s and 65 °C for 30 s. Step 3 (melt curve): 95 °C for 10 s, 65 to 95 °C (increment 0.5 °C) and 95 °C for 5 s. Each qRT-PCR reaction solution for miRNA consisted of the following: 10 μL miRcute Plus miRNA PreMix (2 X) (SYBR and ROX), 0.4 μL PCR Forward Primer (10 μM), 0.5 μL Reverse Primer (10 μM), miRNA First-Strand cDNA template (<200 ng), and Rnase Free dH2O (up to 20 μL). The three steps for the reaction conditions were as follows. Step 1: 95 °C for 15 min. Step 2 (40 cycles): 94 °C for 20 s and 60 °C for 34 s. Step 3 (melt curve): 95 °C for 10 s, 65 to 95 °C (increment 0.5 °C) and 95 °C for 5 s. The amount of RNA used for reverse transcription and qRT-PCR was 1 ug. The relative expressions of miRNA and mRNA were calculated using *U6* and *β-actin* genes as the internal controls, respectively. Furthermore, the 2^−∆∆CT^ method was used for data normalization [32].

### 2.4. Oil Red O Staining and BODIPY Staining

Bovine preadipocytes were induced to differentiate for 10 d, followed by complete removal of the culture medium, and rinsing of the cells with PBS. The cells were then fixed in 4% polyformaldehyde at room temperature for 30 min and stained with fresh Oil Red O (Sangon Biotech, Shanghai, China) working solution for 30 min in a dark room. The cells were removed from the staining solution and washed with PBS. Finally, a light microscope (Olympus, Tokyo, Japan) was used to visualize the cells and to capture the images. On the 4th day of differentiation induction, the culture medium was removed and the cells were fixed in 4% paraformaldehyde for 30 min, as described above. The cells were then stained with BODIPY and incubated for 30 min in the dark. The BODIPY solution was removed, and the cells were washed with PBS. The cells were then stained with DAPI solution (Sangon Biotech) for 30 min in a dark room, after which the DAPI solution was removed and the cells were washed with PBS. Finally, the cells were visualized under a fluorescence microscope (Olympus) and images were captured. Image-Pro Plus 6 was used to analyze the data.

### 2.5. Western Blotting

Extraction of the total proteins from the cells was performed using a RIPA lysis buffer (Beyotime, Shanghai, China) supplemented with 1% phenylmethanesulfonyl fluoride (Beyotime, Shanghai, China) or 1% phosphatase inhibitor buffer (Roche, Basel, Switzerland). Western blot analyses were performed as previously described [33]. The blots were cut prior to hybridization with antibodies during blotting. Immune blots were imaged using chemiluminescent horseradish peroxidase substrate (Millipore, Boston, MA, USA). Images were captured and quantified using the Gel DocTM XR+ System with the Image Lab^TM^ software (Bio-Rad, Hercules, CA, USA). This study involved the use of the following primary antibodies: PPARγ antibody (A00449-2; 1:500; Boster, Sacramento, CA, USA), FABP4 antibody (ab92501; 1:5000; Abcam, Cambridge, UK), SREBF1 antibody (14088-1-AP; 1:400; Proteintech, Rosemount, PA, USA), β-actin antibody (ab8226; 1:5000; Abcam), IRS2 antibody (ab134101; 1:2000; Abcam), Akt antibody (9272; 1:1000; Cell Signaling Technology, Boston, MA, USA), and phospho-Akt (pAkt) antibody (9271; 1:1000; Cell Signaling Technology). Similarly, two secondary antibodies were used in this study (ab150113 and ab150077, 1:5000, Abcam).

### 2.6. Vector Construction

The predicted seed sequence of *IRS2* directly targeted by miR-33a was obtained using TargetScan “http://www.targetscan.org/ (accessed on 10 May 2022)”. The wild-type fragment named IRS2-3′UTR-wt, including the predicted seed sequence, was amplified and inserted into the psiCHECK^TM^-2 vector (Promega, Madison, WI, USA) using the restriction enzymes *Xho* I and *Not* I (Takara, Tokyo, Japan) to construct the recombinant plasmid named psiCHECK^TM^-2-IRS2-3′UTR-wt. Thereafter, the mutation type fragment was designed by changing four of the seed sequence’s nucleotides, constructing the psiCHECK^TM^-2-IRS2-3’UTR-mut as above. Both recombinant plasmids were transformed into competent cells that were coated with plates, while monoclonal colonies were selected to harvest purified plasmids in preparation for the subsequent studies.

### 2.7. Luciferase Activity Assay

Both recombinant plasmids were co-transfected with miR-33a mimic or mimic NC into HEK293A cells cultured in DMEM (Hyclone) supplemented with 10% FBS (PAN-Biotech) and 100 U/mL penicillin/streptomycin when the cell confluence reached approximately 80%. After a culture period of 48 h, we harvested the cells and measured the values for the renilla fluorescence and firefly fluorescence using the dual-luciferase reporter assay system kit (Promega) according to the manufacturer’s instructions. Briefly, cells were lysed using Passive Lysis Buffer (1 X) for 15 min in a 37 °C incubator, and then 20 uL of lysate was subsequently used in the luciferase activity assays. Luciferase Assay Buffer II (50 μL) was added to the lysate and mixed. Subsequently, 50 μL Stop and Glo Substrate (1 X) was added prior to the measurement of the renilla luciferase value. Finally, the ratio of the firefly luciferase value to the renilla luciferase value was calculated. Notably, Passive Lysis Buffer (1 X) and Luciferase Assay Buffer II were incubated at 25 °C and were thoroughly mixed before use.

### 2.8. Statistical Analysis

Statistical analysis involved performing one-way ANOVA and *t*-tests via SPSS (Version 26.0, IBM Corp., Armonk, NY, USA) and the results were expressed as the mean ± SD (n = 3). Group differences were deemed to be statistically significant at *p* < 0.05 or *p* < 0.01. Graphs presented were generated using GraphPad Prism (Version 6.01, San Diego, CA, USA).

## 3. Results

### 3.1. miR-33a Overexpression Inhibits Differentiation of Bovine Preadipocytes

**Figure 1:**
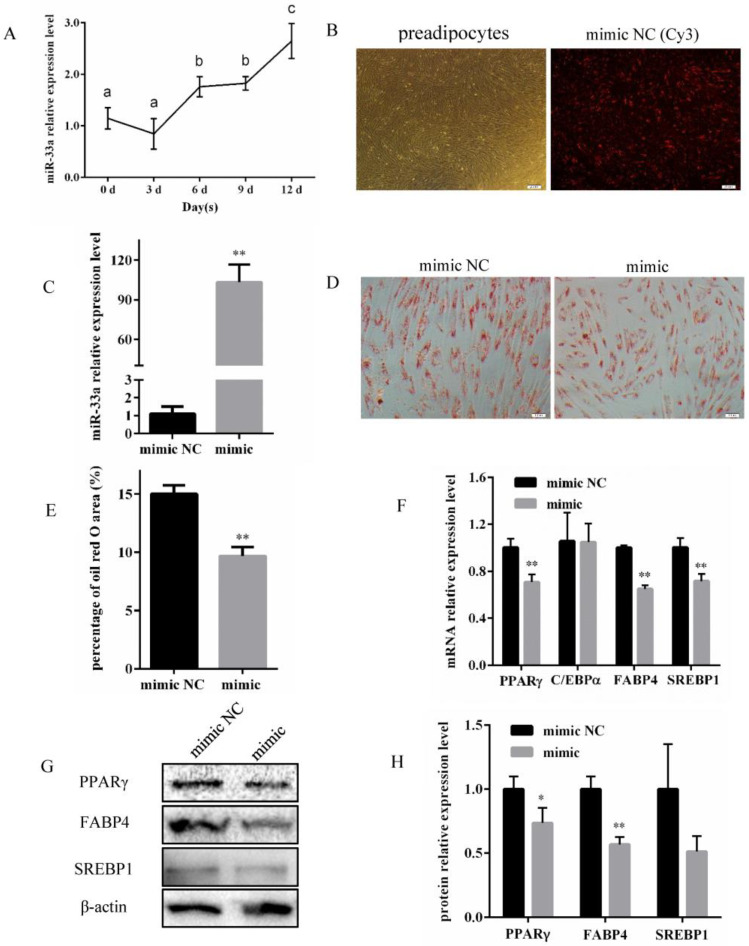
Overexpression of miR-33a inhibits the differentiation of bovine preadipocytes. (**A**) Relative miR-33a expression assessed via qRT-PCR during bovine preadipocyte differentiation at 0, 3, 6, 9, and 12 d. (**B**) Bovine preadipocytes of 100% confluence and the image of preadipocytes transfected with mimic NC (Cy3) for 48 h (Olympus, 200×). (**C**) The efficiency of miR-33a overexpression 48 h post-transfection of bovine preadipocytes with the miR-33a mimic and mimic NC. (**D**) Oil Red O after 10 d of bovine preadipocyte differentiation (Olympus, 200×). (**E**) The percentage of Oil Red O area of miR-33a mimic and mimic NC. (**F**) Relative mRNA expression of the *PPARγ*, *C/EBPα*, *FABP4*, and *SREBP1* genes on the 6th day of bovine preadipocyte differentiation. (**G**) Protein expression analysis of PPARγ, FABP4, and SREBP1 genes by Western blotting on the 6th day of bovine preadipocyte differentiation, with β-actin as an internal reference. (**H**) Statistical data of the protein expression of the PPARγ, FABP4, and SREBP1 genes. Data were expressed as means ± SD (n = 3). Different lowercases among different columns represent *p <* 0.05. * *p* < 0.05, ** *p* < 0.01.

Analysis via qRT-PCR indicated that miR-33a expression was gradually progressively increased during the differentiation of bovine preadipocytes (Figure 1A). Subsequently, bovine preadipocytes were transfected with miR-33a mimic, mimic NC, and mimic NC (Cy3) to investigate whether miR-33a overexpression affected their differentiation. The results indicated that the transfection was successful (Figure 1B) and that the miR-33a expression increased more than 90-fold (Figure 1C) 48 h post-transfection. The lipid content on the 10th day of bovine preadipocyte differentiation after miR-33a overexpression was significantly lower than the control (Figure 1D,E). On the 6th day of bovine preadipocyte differentiation, the expression of *PPARγ*, *FABP4*, and *SREBP1* at mRNA levels decreased significantly (Figure 1F). PPARγ and FABP4 expression decreased significantly at the protein level after overexpressing miR-33a by transfecting the miR-33a mimic, while the protein expression of SREBP1 was also lower, but not significantly (Figure 1G,H). Taken together, these results indicate that miR-33a overexpression inhibits the differentiation of bovine preadipocytes.

### 3.2. Inhibition of miR-33a Expression Promotes Differentiation of Bovine Preadipocytes

**Figure 2:**
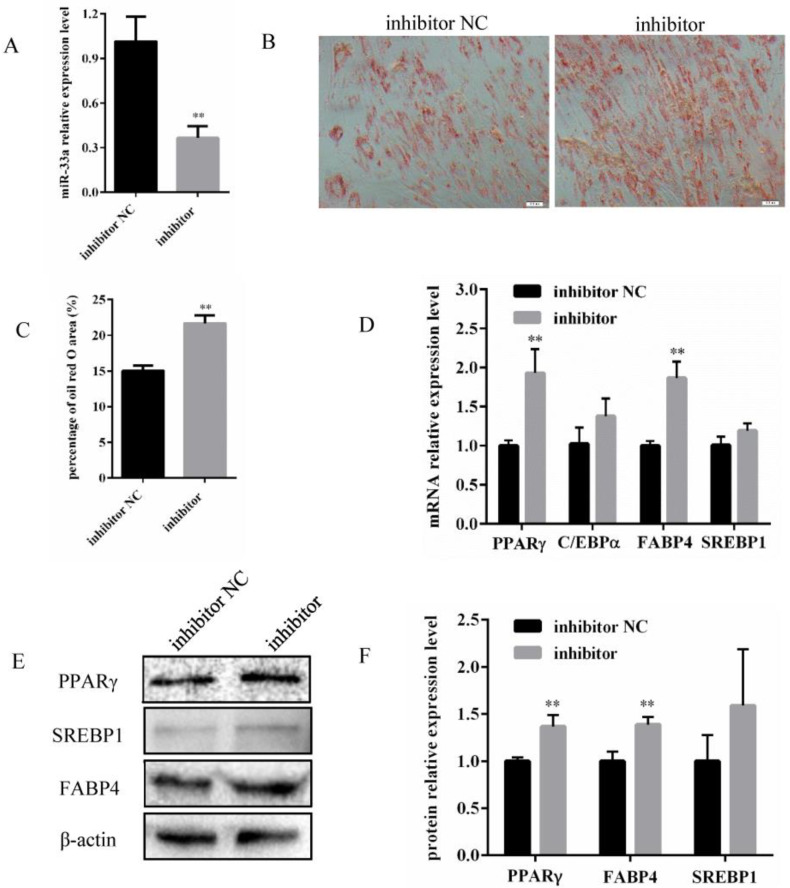
Inhibition of miR-33a expression promotes differentiation of bovine preadipocytes. (**A**) The interference efficiency of miR-33a in bovine preadipocytes 48 h post-transfection with miR-33a inhibitor or inhibitor NC. (**B**) Oil Red O staining at 10 d of bovine preadipocyte differentiation (Olympus, 200×). (**C**) The percentage of Oil Red O area for the miR-33a inhibitor and inhibitor NC. (**D**) Relative mRNA expression of *PPARγ*, *C/EBPα*, *FABP4*, and *SREBP1* genes on the 6th day of bovine preadipocyte differentiation. (**E**) Protein expression analysis of PPARγ, FABP4, and SREBP1 genes by Western blotting with β-actin as an internal reference on the 6th day of bovine preadipocyte differentiation. (**F**) Relative expression levels of PPARγ, FABP4, and SREBP1 proteins. Data are expressed as means ± SD (n = 3). ** *p* < 0.01.

In order to investigate whether the differentiation of bovine preadipocytes was affected by miR-33a inhibition, preadipocytes were transfected with either a miR-33a inhibitor, or an inhibitor NC. After 48 h, miR-33a expression in the miR-33a inhibitor group was significantly reduced relative to the expression in the inhibitor NC group (Figure 2A). Concurrently, Oil Red O staining indicated a significant increase in the lipid content and area of bovine preadipocytes in the inhibitor group compared with the inhibitor NC group on the 10th day of the differentiation (Figure 2B,C). In addition, inhibition of miR-33a expression promoted the mRNA expression (Figure 2D) and protein expression (Figure 2E,F) of PPARγ and FABP4. Taken together, the inhibition of miR-33a promoted the differentiation of bovine preadipocytes.

### 3.3. miR-33a Inhibits IRS2 Expression by Directly Targeting 3′-UTR

**Figure 3:**
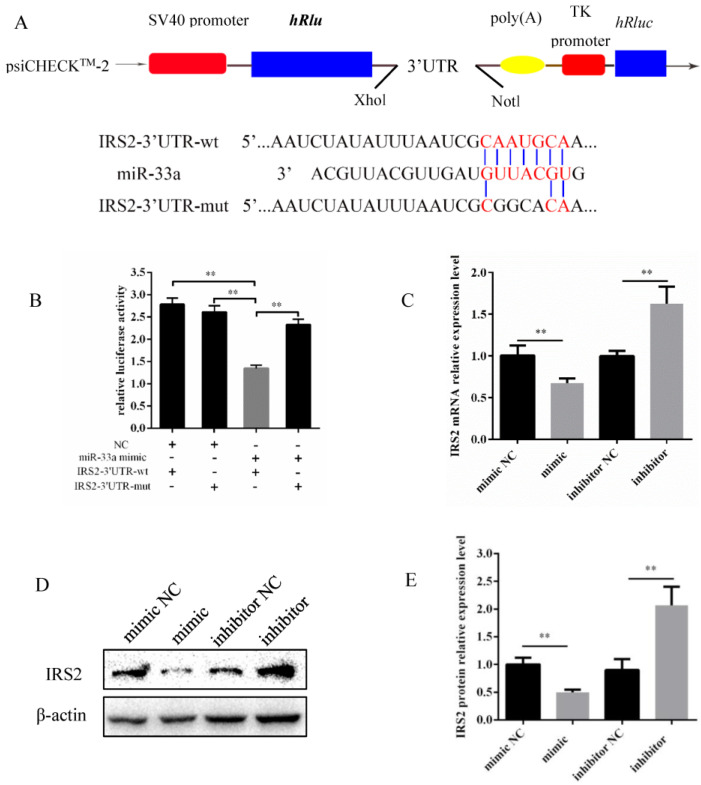
miR-33a suppressed IRS2 expression by directly targeting 3′-UTR. (**A**) Schematic diagram of recombinant plasmids, named psiCHECK-2-IRS2-3′UTR-wt and psiCHECK-2-IRS2-3′UTR-mut, which were constructed using the *Xho* I and *Not* I restriction enzymes. (**B**) Activity of luciferase reporter plasmids recombined with wild-type or mutated IRS2-3′UTR, in HEK293 T cells co-transfected with miR-33a mimic and mimic NC. (**C**) Relative expression of *IRS2* mRNA in bovine preadipocytes 48 h post-transfection with the miR-33a mimic, mimic NC, inhibitor, and inhibitor NC, respectively. (**D**) Relative protein expression of IRS2 in bovine preadipocytes 48 h post-transfection with the miR-33a mimic, mimic NC, inhibitor, and inhibitor NC, respectively. β-actin was used as an internal reference. (**E**) Relative expression of IRS2 protein (plot generated via Image Lab). Data are expressed as means ± SD (n = 3). ** *p* < 0.01.

Targetscan was used to identify *IRS2* as a predicted target gene for miR-33a. To investigate whether miR-33a directly targets *IRS2*, we constructed psiCHECK-2 recombinant vectors integrating IRS2 3′UTR wild-type and mutant seed sequences, named psiCHECK-2-IRS2-3′UTR-wt and psiCHECK-2-IRS2-3’UTR-mut, respectively (Figure 3A). Subsequently, these recombinant vectors were transfected into HEK293A cells with miR-33a mimic and mimic NC. The results indicate that miR-33a mimic significantly inhibited the luciferase activity of psiCHECK-2-IRS2-3′UTR-wt when compared with other groups (Figure 3B). Moreover, the overexpression of miR-33a downregulated the *IRS2* expression, and conversely, the inhibition of miR-33a led to the upregulation of *IRS2* both at the mRNA and protein levels of IRS2 in bovine preadipocytes (Figure 3C–E). Therefore, these results indicate that miR-33a directly targets *IRS2* to inhibit its expression at the mRNA and protein levels.

### 3.4. miR-33a Regulated the Activity of the IRS2–Akt Pathway in Preadipocyte Differentiation

**Figure 4:**
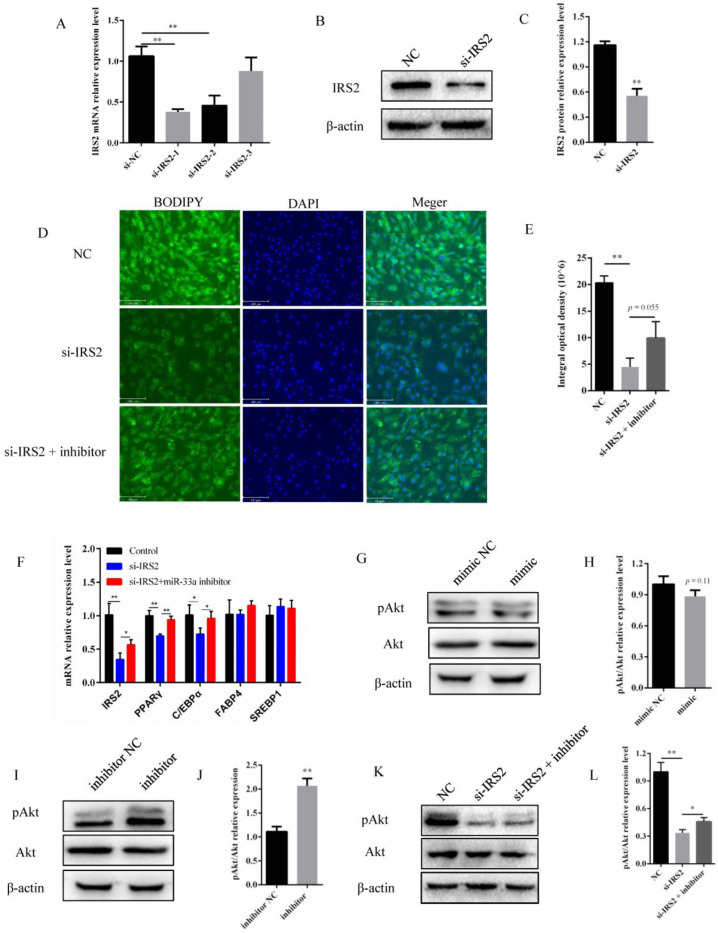
miR-33a regulates the activity of the IRS2–Akt pathway in preadipocyte differentiation. (**A**) The interference efficiency of three siRNAs on *IRS2*. (**B**) Relative expression of IRS2 protein after 48 h of transfection with si-IRS2 and NC. (**C**) Expression levels of IRS2 protein (**D**) BODIPY staining on the 4th day of bovine preadipocyte differentiation treatment with NC or si-IRS2 or si-IRS2 with miR-33a inhibitor (Olympus, 200×). (**E**) The integral optical density of BODIPY fluorescence (Olympus, 200×). (**F**) Relative expression of *IRS2*, *PPARγ*, *C/EBPα*, *FABP4*, and *SREBP1* mRNA on the 6th day of bovine preadipocyte differentiation transfected with the miR-33a mimic and mimic NC. (**G**) Western blot analysis of pAkt and Akt protein expression on the 6th day of bovine preadipocyte differentiation after transfection with the miR-33a mimic and mimic NC. Furthermore, β-actin was used as an internal reference. (**H**) Protein expression of pAkt/Akt. (**I**) Western blot analysis of pAkt and Akt protein expression on the 6th day of bovine preadipocyte differentiation after transfection with the miR-33a inhibitor and inhibitor NC. (**J**) Statistical data of the relative protein expression of pAkt/Akt. (**K**) Protein expression analysis of pAkt and Akt genes by Western blotting after transfection with NC, si-IRS2, and the mix of si-IRS2 and miR-33a inhibitor. (**L**) Relative expression of pAkt/Akt protein. Data are expressed as means ± SD (n = 3). * *p* < 0.05, ** *p* < 0.01.

To investigate whether *IRS2* influenced the differentiation of preadipocytes in cattle, three small interfering RNAs (siRNAs) targeting *IRS2* were synthesized and transfected into bovine preadipocytes. The subsequent analysis indicated that two of these siRNAs had a significant interference efficiency, of which the one with the highest interference efficiency, i.e., si-IRS2-1, (named si-IRS2 hereafter), was used in the subsequent assays. The si-IRS2 siRNA reduced the *IRS2* expression by approximately 60% at the mRNA level, and by approximately 50% at the protein level (Figure 4A–C). The BODIPY staining results showed that interference with *IRS2* could significantly inhibit the lipid content, while inhibition of miR-33a appeared to partially rescue the damage to the lipid content caused by si-IRS2 (Figure 4D,E). Downregulation of *IRS2* was found to inhibit *PPARγ* and *C/EBPα* mRNA expression, and, furthermore, miR-33a inhibition was found to partially rescue the expression of *IRS2*, *PPARγ*, and *C/EBPα* mRNA (Figure 4F). Collectively, these results indicate that both miR-33a and *IRS2* contribute to regulating bovine preadipocyte differentiation.

Previous studies have identified *IRS2* as being involved in regulating the Akt pathway signaling [34,35,36]. Based on this, we hypothesized that miR-33a may downregulate Akt signaling in bovine preadipocytes by targeting *IRS2*. In order to investigate this, bovine preadipocytes were transfected with a miR-33a mimic, mimic NC, miR-33a inhibitor, and inhibitor NC. Western blot analysis indicated that miR-33a overexpression decreased the phosphorylation level of Akt, and, conversely, miR-33a inhibition increased the phosphorylation level of Akt (Figure 4G–J). In addition, Western blot analysis of bovine preadipocytes co-transfected with a miR-33a inhibitor indicated that it partially rescued the *IRS2* mediated downregulation of Akt phosphorylation (Figure 4K,L). Taken together, these results indicate that miR-33a contributes to the inhibition of the differentiation of bovine preadipocytes, at least in part by inhibiting the IRS2–Akt pathway.

## 4. Discussion

Preadipocyte differentiation is a multistep process that includes cell fate determination and clonal expansion, and ultimately results in terminal differentiation. In recent decades, several studies have attempted to identify miRNAs that target crucial genes involved in modulating preadipocyte differentiation at different stages during adipogenesis. Several miRNAs that are differentially expressed during adipocyte differentiation have been shown to regulate adipogenesis [37]. In agreement with such findings, this study confirmed that miR-33a expression was altered during different stages of differentiation of bovine preadipocytes (Figure 1A). The overexpression of miR-33a was found to inhibit the differentiation of preadipocytes and lipid accumulation within them (Figure 1). In contrast, the inhibition of miR-33a promoted the differentiation and lipid accumulation of preadipocytes (Figure 2). In ovine adipose-derived stromal vascular fraction cells, miR-33a could also impair adipogenic differentiation by targeting *SIRT6* gene [38]. In bovine mammary epithelial cells, miR-33a expression increased the unsaturated fatty acid content and promoted triglyceride synthesis [39]. In addition, miR-33a inhibited the differentiation of human adipose-derived mesenchymal stem cells towards urothelial cells by targeting β-catenin and transforming growth factor receptors [40]. This study revealed that miR-33a might play a crucial role in adipose differentiation. Contrary to our findings, miR-33a expression in white adipose tissue was previously found to be significantly reduced in obese mice when compared with lean mice [41]. Some researchers have shown that it is expressed differently in the intramuscular fat tissue of bulls and steers and is less expressed in steers than in bulls [22]. Castrating bulls has been found to significantly increase the deposition of intramuscular fat in the longissimus muscle, also known as marbling, leading to improved beef quality and price [42]. Therefore, studying the role of miR-33a in preadipocyte differentiation might have substantial implications for human obesity and the meat-quality traits of livestock, especially cattle.

At present, the dual-luciferase reporter assay is frequently relied upon in order to confirm the targeting relationship between a specific miRNA and a predicted gene [43]. The same assay was used to identify *IRS2* as a direct target of miR-33a in our study (Figure 3). Previous murine studies have shown that miR-33a directly targeted *IRS2* and regulated pAkt protein levels [44]. Meanwhile, some studies have revealed that *IRS2* is a target gene of miR-33a in ovine preadipocytes and regulates its differentiation. Our study also demonstrated this by detecting the levels of adipocyte-specific genes and lipid accumulation (Figure 4). In goat mammary epithelial cells, *IRS2* is targeted by miR-181b, partially rescuing the decrease in triglyceride levels and milk fat droplet accumulation induced by miR-181b [45]. Furthermore, human bone marrow-derived mesenchymal stem cells (hMSCs) are multipotent stem cells with an adipogenic capacity [46]. Previous studies have shown that the knockdown of *IRS2*, directly targeted by miR-431, inhibited hMSCs differentiation into adipocytes [47]. *IRS1* and *IRS2* are critical downstream regulators of lipid metabolism [48]. Interestingly, *IRS1* and *IRS2* knockdown in 3T3-L1 murine preadipocytes impaired adipocyte differentiation and lipid accumulation; however, their knockdown in mature adipocytes did not have a similar effect on the cellular lipid content [29]. These findings indicate a potentially crucial role for *IRS2* in preadipocyte differentiation and lipid metabolism.

Akt serine/threonine kinase (Akt) is among the most crucial genes involved in PI3K–Akt signaling that regulates the proliferation, differentiation, and apoptosis of preadipocytes [49,50]. The Ser473 phosphorylation site of Akt is an important segment in regulating the PI3K–Akt signaling pathway [51]. It is well known that *IRS2* regulates the phosphorylation site of Akt at Ser473, which was also observed in our study (Figure 4). Furthermore, the miR-33a inhibitor rescued the decrease in lipid content, adipocyte differentiation marker gene expression, and Akt phosphorylation levels induced by si-IRS2 (Figure 4). In conclusion, our results indicate that miR-33a contributes to regulating the differentiation of bovine preadipocytes. In addition, miR-33a may regulate the differentiation of bovine preadipocytes through the IRS2–Akt pathway. In the long term, these findings might contribute to the development of novel and alternative opportunities to improve beef quality.

## Data Availability

The data supporting the present study are available from the corresponding author upon reasonable request.
